# The management of acute knee dislocations: A global survey of orthopaedic surgeons’ strategies

**DOI:** 10.1051/sicotj/2021017

**Published:** 2021-03-26

**Authors:** Santa-Marie Venter, Roopam Dey, Vikas Khanduja, Richard PB von Bormann, Michael Held

**Affiliations:** 1 Department of Orthopedic Surgery, Groote Schuur Hospital, Orthopedic Research Unit, University of Cape Town Cape Town 7925 South Africa; 2 Consultant Orthopedic Surgeon, Addenbrooke’s Hospital, Cambridge, University of Cambridge Cambridge CB2 2QQ United Kingdom; 3 Department of Human Biology, Division of Biomedical Engineering, University of Cape Town Cape Town 7925 South Africa; 4 Cape Town Sports and Orthopaedic Clinic, Christian Barnard Memorial Hospital Cape Town 8001 South Africa

**Keywords:** Multiligament knee injury, Acute knee dislocation, Management knee dislocation

## Abstract

*Purpose*: Great variety and controversies surround the management strategies of acute multiligament knee injuries (aMKLIs) and no established guidelines exist for resource-limited practices. The aim of this study was to compare the management approach of acute knee dislocations (AKDs) by orthopedic surgeons from nations with different economic status. *Methods*: This descriptive cross-sectional scenario-based survey compares different management strategies for aMLKIs of surgeons in developed economic nations (DEN) and emerging markets and developing nations (EMDN). The main areas of focus were operative versus non-operative management, timing and staging of surgery, graft choice and vascular assessment strategies. The members of the Societe Internationale de Chirurgie Orthopedique et de Traumatologie (SICOT) were approached to participate and information was collected regarding their demographics, experience, hospital setting and management strategies of aMLKIs. These were analyzed after categorizing participants into DEN and EMDN based on the gross domestic product (GDP) per capita. *Results*: One-hundred and thirty-eight orthopedic surgeons from 47 countries participated in this study, 67 from DEN and 71 (51.4%) from EMDN. DEN surgeons had more years of experience and were older (*p* < 0.05). Surgeons from EMDN mostly worked in public sector hospitals, were general orthopedic surgeons and treated patients from a low-income background. They preferred conservative management and delayed reconstruction with autograft (*p* < 0.05) if surgery was necessary. Surgeons from DEN favored early, single stage arthroscopic ligament reconstruction. Selective Computerized Tomography Angiography (CTA) was the most preferred choice of arterial examination for both groups. Significantly more EMDN surgeons preferred clinical examination (*p* < 0.05) and duplex doppler scanning (*p* < 0.05) compared to DEN surgeons. More surgeons from EMDN did not have access to a physiotherapist for their patients. *Conclusions*: Treatment of aMLKIs vary significantly based on the economic status of the country. Surgeons from DEN prefer early, single stage arthroscopic ligament reconstruction, while conservative management is favored in EMDN. Ligament surgery in EMDN is often delayed and staged. EMDN respondents utilize duplex doppler scanning and clinical examination more readily in their vascular assessment of aMLKIs. These findings highlight very distinct approaches to MLKIs in low-resource settings which are often neglected when guidelines are generated.

## Introduction

Acute multiligament knee injuries (aMLKIs) are uncommon injuries, however, if not recognized and managed appropriately, they can have devastating consequences [1]. The popliteal artery is injured in 1.6% [2] to 40% [3] of cases and vascular assessment forms a crucial, yet controversial part of the initial assessment. Ligament reconstruction can be performed acutely (<3 weeks), delayed (>3 weeks), or it can be staged [4]. Conservative treatment with bracing is reserved for certain compromised patients and if access to surgical care is restricted [5].

The prognosis following an aMLKI depends on many factors such as the velocity of injury [6], associated neurovascular damage [7], treatment methods, rehabilitation [8], and more recently, obesity was also found to play a role [9]. The treatment of knee dislocations has been inconsistent, although surgical treatment has become the preferred option [10] and high-volume centers in the developed world recommend early single-stage arthroscopic ligament reconstruction with auto – or allografts [11]. For vascular assessment, selective angiography is regarded by many as the modality of choice [12]. Yet, for resource-constrained settings in low-income countries, there are no evidence-based guidelines that are adapted to local challenges, such as access to surgical time, sub-specialist surgeons, arthroscopic equipment, allograft, and physiotherapy.

The aim of our study was therefore to compare the management approach of aMLKIs by orthopedic surgeons from developed economic nations (DEN) and emerging markets and developing nations (EMDN), specific to resources available. Given the resource-constraints of hospitals and the socio-economic circumstances of patients in EMDN, we hypothesize that the approach of orthopedic surgeons towards aMLKIs would be different compared to surgeons from DEN.

## Materials and methods

All procedures performed in studies involving human participants were in accordance with the ethical standards of the institutional and/or national research committee and with the 1964 Helsinki declaration and its later amendments or comparable ethical standards. This study was approved by the Human Research Ethics Committee of a tertiary academic government hospital (HREC REF 050/2018). Informed consent was obtained from all surgeons participating in the survey.

This descriptive cross-sectional questionnaire-based survey was designed to assess the treatment choices made by orthopedic surgeons around the world.

### Questionnaire development

The questionnaire was generated by the core research team, based on propositions made by subspecialist knee surgeons during a focus group interview of knee surgeons. This was then sent to a group of knee surgeons for feedback. After adjustments and approval by the research team, the questionnaire was finalized (Appendix). Before answering the questions, every participant provided informed consent. The questionnaire consisted of 26 questions: 12 (46.2%) multiple-choice questions, 4 binary questions, and 10 subjective questions of which 4 were optional depending on the previous response. Questionnaires took approximately 5 min to complete.

### Survey population

This questionnaire was then sent to members of the Societe Internationale de Chirurgie Orthopedique et de Traumatologie (SICOT) via email with three monthly reminders from July 2019 – September 2019. All completed questionnaires were included. Excluded were double entries or incomplete submissions. Study data were anonymously collected and managed using Research Electronic Data Capture (REDCap). REDCap is a secure, web-based software platform designed to support data capture for research studies, providing (1) an intuitive interface for validated data capture; (2) audit trails for tracking data manipulation and export procedures; (3) automated export procedures for seamless data downloads to common statistical packages, and (4) procedures for data integration and interoperability with external sources.

### Data analysis

**Figure 1:**
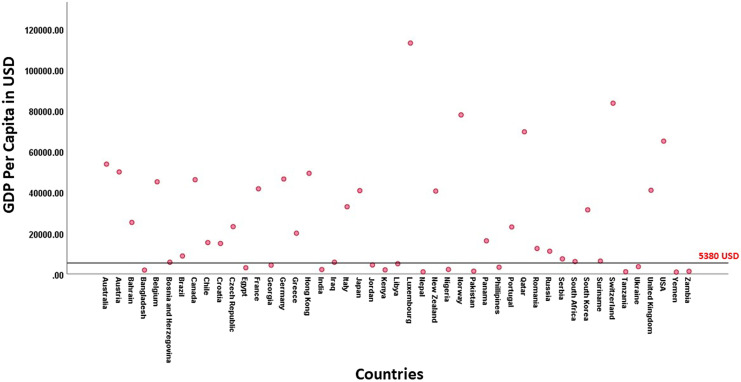
The gross domestic product (GDP) of the countries from which the study’s surgeon population belonged. The cut-off GDP was $5380 shown by the black line.

Responses to questionnaires were analyzed with reference to the management approaches of aMLKIs, and the responder nation’s socio-economic status. Participating surgeons were divided into two groups based on their country’s Gross Domestic Product (GDP) per-capita: DEN and EMDN. The cut-off GDP was set equal to the average GDP of all the EMDN countries in the world, pre-COVID-19 pandemic, as reported by the International Monetary Fund ($5380) [13]. Any country below this limit was grouped as EMDN (Figure 1).

Demographic data recorded included the age, gender, years of experience, and level of specialization of the participating surgeons. The continent and country of residence, socio-economic status of patients and sector of service were also included. This information was collected to judge the patients’ access to treatment. The number of anterior cruciate ligament (ACL) injuries and MLKIs treated per year, as well as access to arthroscopy equipment, magnetic resonance imaging (MRI), and physiotherapy, were recorded.

### Statistical analysis

Responses from surgeons hailing from the same country were added and reported as percentages. Mean and standard deviation was calculated for the surgeon’s age and experience. The data analyses were performed in IBM SPSS Statistics v.26 (Armonk, NY: IBM Corp). Non-parametric tests for significance, the Mann-Whitney U test was used to compare the responses from the EMDN and DEN groups. The level of significance was set at *p* < 0.05.

## Results

### Surgeon demographics

One-hundred and thirty-eight participants, from 47 countries, submitted their responses. 32 countries (67 surgeons, 48.5%) were DEN and the remaining 15 countries (71 surgeons, 51.4%) were identified as EMDN. The surgeons’ age, gender, and experience are presented in Table 1. DEN surgeons were significantly older (*p* < 0.05) and had more years of surgical experience (*p* < 0.05) compared to participating surgeons in the EMDN.

**Table 1:** Demographic details of surgeons.

Groups	Average age (range) in years	Male:Female	Average experience (range) in years
Overall	47.18 (31–73)	131:7	15.02 (01–40)
DEN	50.02 (32–73)	61:6	17.53 (02–40)
EMDN	41.13 (31–67)	70:1	9.67 (01–35)

### Hospital sector and patient socioeconomic status

**Supplementary Figure 2:**
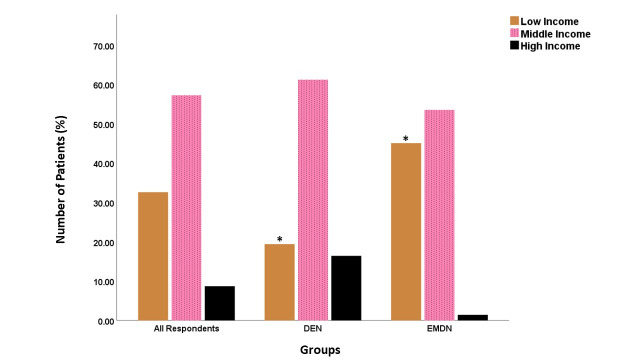
Patient’s socio-economic status as reported by the participating surgeons. Significant differences are denoted by *.

The number of surgeons working in private sectors was higher (*n* = 28, 41.8%, *p* > 0.05) for the DEN group, while a significantly higher number of surgeons (*n* = 32, 45.1%, *p* < 0.05) worked in the public sector hospitals in the EMDN group (Supplementary Fig. 1). Twenty surgeons (29.9%) who completed the questionnaire from the DEN group were sub-specialized knee surgeons, compared to only 9 (12.7%) surgeons from the EMDN group (*p* > 0.05). Seventy nine of the overall respondents (57.3%) reported that their patients belonged to the middle-income category (Supplementary Fig. 2). Surgeons in the EMDN countries treated a significantly higher number of patients from the low-income bracket (*n* = 32, 45.1%) compared to only (*n* = 11, 16.4%, *p* < 0.05) of surgeons from the DEN countries, who treated more high-income (16.4%) patients compared to surgeons in the EMDN group (1.4%, *p* > 0.05).

### Annual surgery load

The average number of ACL surgeries performed across both the groups (DEN: 62.06; EMDN: 68.42), were consistently higher than the MLKI surgeries performed (DEN: 9.99; EMDN: 13.52). The ratio of these surgeries varied significantly between the DEN (ratio = 6.85) and EMDN (ratio = 4.15, *p* < 0.05) groups.

### Management strategy and grafts

**Figure 2:**
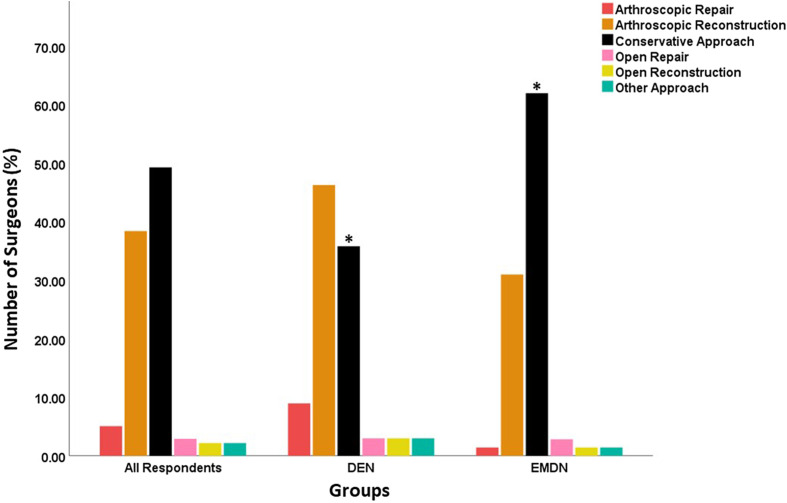
The different surgical approaches used by the participating surgeons to treat aMLKIs. Significant differences are denoted by *.

**Figure 3:**
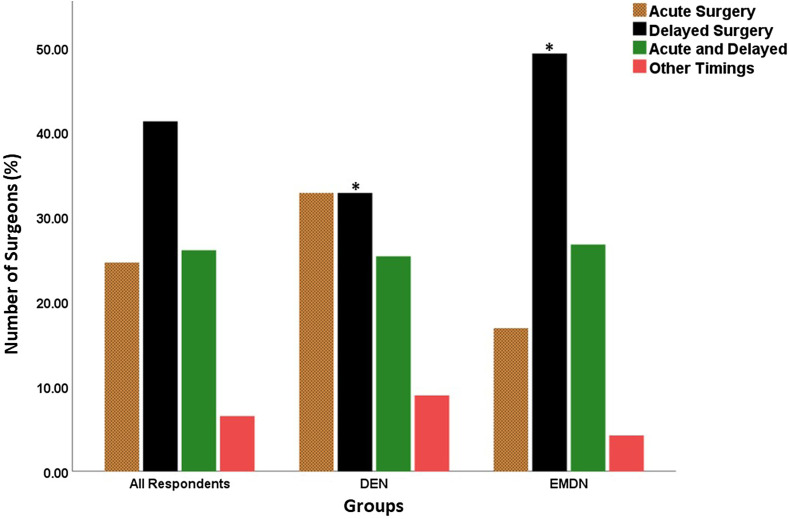
Timing of the aMLKI surgeries as reported by the participating surgeons. Significant differences are denoted by *.

The acute management strategy of MLKIs varied between surgeons from DEN and EMDN countries. Arthroscopic reconstruction of cruciate ligaments was preferred by surgeons from the DEN group (*n* = 31, 46.3%), while EMDN participants favored conservative management (*n* = 44, 62%, *p* < 0.05) (Figure 2). DEN Surgeons preferred acute and delayed surgery equally (*n* = 22; 32.8%), while a significantly higher number of surgeons from EMDN preferred delayed surgery (*n* = 35; 49.3%; *p* < 0.05) (Figure 3). Autograft was preferred significantly more by the surgeons in the EMDN group (*n* = 56, 78.9%) compared to surgeons from the DEN group (*n* = 38, 56.7%, *p* < 0.05). More EMDN surgeons (*n* = 46, 64.8%) do not use allograft compared to DEN participants (*n* = 23, 34.3%, *p* < 0.05).

### Vascular examination and access to physiotherapy

**Figure 4:**
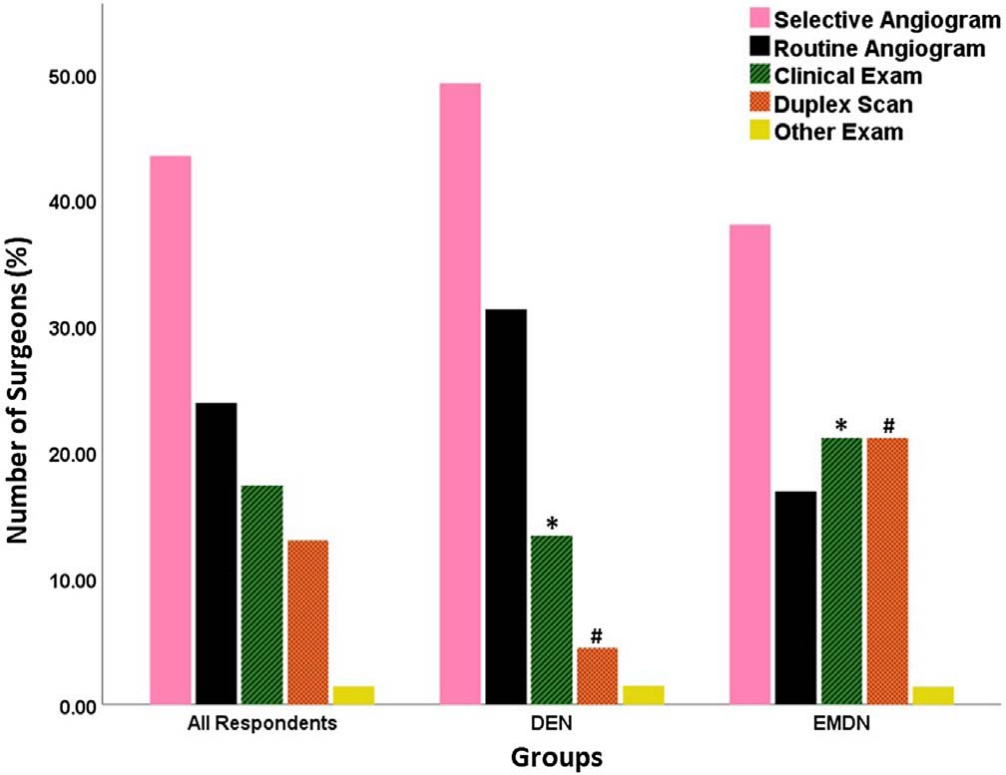
Various vascular examinations that were reported to be performed by the participating surgeons. Significant differences are denoted by * and #.

**Figure 5:**
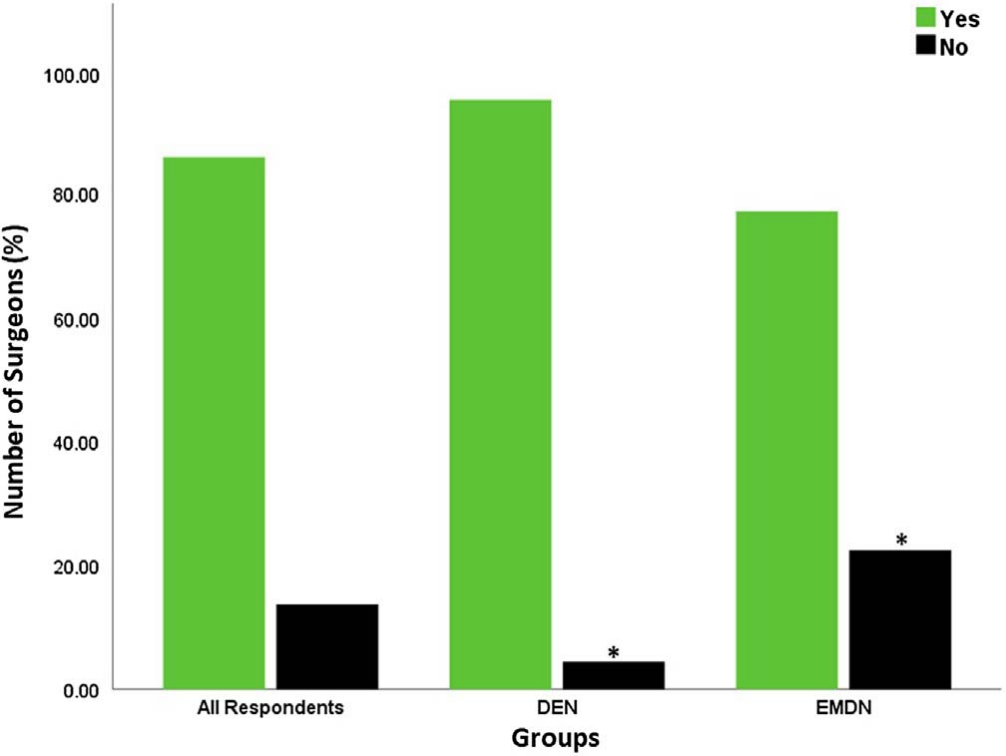
Access to physiotherapy plans as reported by the participating surgeons. Significant differences are denoted by *.

Selective Computed Tomography Angiography (CTA) was the preferred choice of vascular examination to exclude vascular injuries (Figure 4) for both the DEN (*n* = 33, 49.3%) and the EMDN (*n* = 27, 38%) groups. A significantly higher number of surgeons from the EMDN group preferred clinical examination (*n* = 15, 21.1%, *p* < 0.05) and duplex doppler scanning (*n* = 15, 21.1%) compared to surgeons from DEN group (*n* = 9, 13.4%, *p* < 0.05) and (*n* = 3, 4.5%, *p* < 0.05) respectively. A significantly higher number of surgeons from the EMDN group (*n* = 16, 22.5%) had no access to physiotherapists compared to the surgeons from the DEN group (*n* = 3, 4.5%, *p* < 0.05) (Figure 5).

## Discussion

The most important finding of the present study was the significant differences in management strategies of MLKIs when comparing DEN to EMDN. Other significant differences include the practice setting, experience, and specialization of participating surgeons.

Participating EMDN surgeons were younger, had fewer years of experience, worked more commonly in the public sector, and had a lower proportion of subspecialists. They also reported treating a higher portion of patients from low socioeconomic backgrounds.

Limited training posts in EMDN could contribute to the lower number of qualified subspecialists. Patients from lower socio-economic backgrounds, an increased workload, and a lack of resources in EMDN result in an increased need for orthopedic surgeons to work in the public sector. Research regarding orthopedic surgeon density has revealed that in DEN there are more orthopedic surgeons available per 100,000 population [14] than in EMDN. It was also noted that the number of training posts available per 100,000 is much higher in DEN when compared to EMDN.

The differing levels of surgeon experience between the two groups could have influenced management strategies in MLKI. As such, early, single-stage arthroscopic ligament reconstruction is recommended for knee dislocations by DEN centers [15], but conservative management is favored by most surgeons in EMDN. Furthermore, in EMDN, surgery is often delayed when indicated and is more commonly staged and performed via open cruciate surgery (Figure 4).

This might be due to the lack of theater access and resources as well as an increased trauma load [16]. To date, there is no high-level evidence to promote operative over conservative management, but non-operative management is usually reserved for patients unfit for surgery or in settings with severe resource constraints. A meta-analysis of retrospective studies with low levels of evidence compared operative to conservative treatment of MLKIs in 206 patients [17]. The surgical group had a 15° more range of motion (ROM) when compared to the non-surgical group. There were otherwise no significant differences in stability, return to sport, or work. Functional rehabilitation was noted to be the most important prognostic factor. A recently published report of sports-related MLKIs promotes single-stage anatomic knee ligament reconstructions with immediate post-operative rehabilitation as this yielded significantly improved outcomes. This is in line with the current trend of surgical management, yet arthrofibrosis had still been developed by 9.3% of these patients, who required further surgery [15].

Furthermore, physiotherapy services were not readily accessible to 16 surgeons from the EMDN group (22.5%). This is likely due to remote and/or rural locations with rationing of services (service prioritization) [18], compounded by public transport challenges that limit patient accessibility. With limited physiotherapy services, many surgeons will also likely favor delayed or staged surgery as options for the treatment of aMLKIs in EMDN to avoid post-operative stiffness.

Our survey demonstrated that delayed and staged reconstruction of aMLKIs has important roles for all participants, especially in countries with limited resources (*p* = 0.03). DEN surgeons reported an equal preference for acute surgery and delayed surgery, while more surgeons from the EMDN group preferred delayed surgery (Figure 5). The reason for this could be available resources in DEN, where lack of theater access, surgeon availability, limited access to physiotherapy, and poor patient compliance are less common.

A meta-analysis by Levy et al. [19] suggested that early operative treatment of MLKIs improved functional and clinical outcomes when compared to delayed surgery with similar outcomes in knee stability, ROM, or activities of daily living. Another systematic review [20] found equivalent outcomes in terms of knee stability, but acute surgery was strongly associated with ROM deficits. Similarly, a more recent review found that acute surgery increases the risk of requiring manipulation under anesthesia or arthrolysis [21].

Regarding graft choice, surgeons from all socio-economic settings preferred autograft for surgical ligament reconstruction, although there was a higher use of allograft in DEN. Proportionally more surgeons preferred not to use allograft in the EMDN (64.79%) compared to the DEN group (34.33%, *p* = 0.009).

For allograft, factors such as availability, cost [22], and the potential for disease transmission can be the reasons for the decreased use in EMDN. A recent systemic review indicates that autografts lead to better outcomes, are more cost-effective, and should be the first choice [23]. Using allograft does however save time and avoids the potential for donor site morbidity [24]. The decision of graft choice ultimately depends on the number of ligaments requiring reconstruction or augmentation, graft availability, surgeon preference, patient-specific factors, and the chosen surgical technique for reconstruction. Concomitant neurovascular injuries and choice of surgical approach should also be considered when choosing graft options.

Regarding workup for vascular compromise in MLKIs, selective CTA is the gold standard used by many centers [25, 26]. This was also reflected in our study as the preferred choice of arterial examination in both groups (DEN: 49.6%; EMDN: 38%). However, more surgeons in resource-limited settings utilized duplex doppler scanning and clinical examination than their colleagues in developed countries.

Routine CTA played a larger role for DEN (31.3%) compared to EMDN surgeons (16.9%).

The need for arteriography in MLKIs was promoted by Jones et al. [27] in 1979 who deemed clinical examination unreliable. This was disproven by a subsequent study arguing that vascular examination is acceptable to screen patients for the need of “selective” arteriography [28]. This data was utilized by Stannard et al. [29], who developed and tested a widely used protocol of selective angiography. According to our survey, EMDN surgeons also follow this philosophy, although repeated clinical examinations are time-consuming and need well-trained staff. This can be challenging in hospitals with resource restrictions and a large trauma burden. A recent systematic review illustrated the lack of consensus among practitioners regarding the diagnostic and treatment algorithm for vascular injury in MLKIs [12]. A heightened clinical suspicion of vascular injury should be had by surgeons, and they should err on the side of caution to exclude this diagnosis with the best possible means available.

Our study had some limitations. The focus group interviews for the questionnaire did not involve low-volume surgeons, which might have excluded possible treatment options. Yet, it was developed through a formal process, tested, and adjusted before its use.

The questionnaire was completed by surgeons from a wide geographic footprint including Asia, Africa, and Europe. However, we realize that non-participating countries might treat MLKIs differently. We, therefore, included the DEN and EMDN categories to create applicability for non-participating countries with similar socioeconomic circumstances. Also, more options to describe patient profiles (i.e. skeletal immaturity, athletes, elderly, obese) or specific resources (i.e. frequency and extent of physiotherapy) could have provided more insight into the various treatment philosophies.

## Conclusion

This study showed that surgeons from EMDN preferred to treat knee dislocations conservatively when compared to their colleagues in DEN. They also favored delayed and/or staged surgery when the decision was made to surgically intervene. EMDN surgeons also utilized clinical arterial examination and duplex doppler scanning more readily to assess vascular status in MLKIs. These findings highlight very distinct approaches to MLKIs in low-resource settings which are often neglected when guidelines are generated. Clinical studies should be pursued in order to generate more recommendations and evidence regarding the conservative, delayed, and staged surgical treatment of MLKIs in overburdened developing countries with poor resources.

## Declarations

Funding: None

Ethics approval: HREC 647/2018

## Conflicts of interest

Santa-Marie Venter: The author has no conflict of interests to declare.

Roopam Dey: The author has no conflict of interests to declare.

Vikas Khanduja: Educational consultant: Smith & Nephew and Arthtrex.

R von Bormann: The author has no conflict of interests to declare.

Michael Held: The author has no conflict of interests to declare.

## Availability of data and material

### Author’s contribution

Santa-Marie Venter: Protocol writing, research and ethics approval, data analysis and interpretation, article writing, manuscript revision, final article submission.

Roopam Dey: Statistical analysis, result generation, manuscript revision.

Vikas Khanduja: Conceptualisation of study, data collection.

Richard von Bormann: Conceptualisation of study, data collection.

Michael Held: Conceptualisation of study, data collection, editing, manuscript revision.

## Supplementary Material

The Supplementary material of this article is available at https://www.sicot-j.org/10.1051/sicotj/2021017/olm**Supplementary Figure 1:**
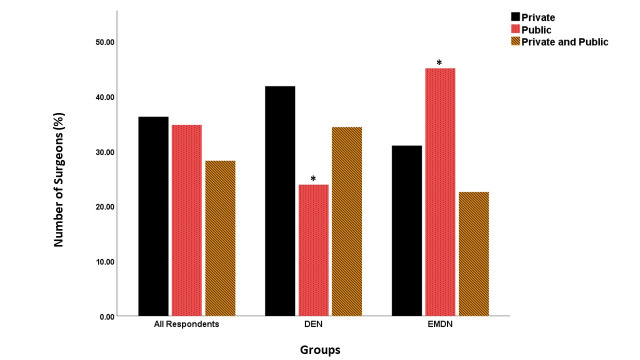
The clinical sectors where the responders operated. Significant differences are denoted by *.

## Appendix

### Consent

Do you agree to anonymously participate in this survey based analysis and grant permission that the results of this survey may be used in a scientific study?

Yes/No

#### Surgeon Profile

Age Range – Decades Region – Continents – Type of Hospital – University, Private, Secondary Care Hospital Sub-specialty.

#### Profile of Practice

Do you treat sporting injuries of the knee on a regular basis?

How many cases of ligament injuries in the knee do you see per annum?

How many multiligament knee injuries do you treat per year?

Do you have access to arthroscopic equipment? Do you have access to an MRI scan for most (80%) of your patients?

#### Management

How do you assess for arterial perfusion in acute multiligament knee injuries?

- Clinical examination only
- Selective angiography (only if physical exam is abnormal)
- Routine angiography
- Duplex scan
- Other

How do you manage multi-ligament injuries in the acute phase (upto 3 weeks)?

- Conservative
- Arthroscopic Repair & Reconstruction
- Open Repair & Reconstruction
- Other

How often do you treat multi-ligament knee injuries conservatively ONLY in your practice?

- Never
- Sometimes
- Often
- Always

For those patients who are treated surgically, do you operate acutely (within 3 weeks) or delayed (after 3 weeks)?

What is your primary choice for reconstruction of torn ligaments (including posterolateral corner) in multiligament knee injuries?

- Autograft
- Allograft
- ACL Repair
- Internal Bracing

For those patients who are managed conservatively, what do you place them in?

- Knee brace
- Cast
- Other

And in what position and for how long? Please describe your protocol?

Do you have access to a dedicated physiotherapy programme post-operatively?

What outcome measures do you use for assessment of multilligament injuries?

What percentage of your patients return to preinjury level of sports?

- 0–25%
- 25–50%
- 50–75%
- 75–100%

What percentage of your patients return to preinjury level of work?

- 0–25%
- 25–50%
- 50–75%
- 75–100%

What is the incidence of stiffness in your practice following conservative or surgical management of muli-ligament injuries?

What is the incidence of complications in your practice following conservative or surgical management of muli-ligament injuries?

## References

[1] Boyce RH , Singh K , Obremskey WT (2015) Acute management of traumatic knee dislocations for the generalist. J Am Acad Orthop Surg 23, 761–768. 26493970 10.5435/JAAOS-D-14-00349

[2] Sillanpää PJ , Kannus P , Niemi ST , Rolf C , Felländer-Tsai L , Mattila VM (2014) Incidence of knee dislocation and concomitant vascular injury requiring surgery: A nationwide study. J Trauma Acute Care Surg 76, 715–719. 24553539 10.1097/TA.0000000000000136

[3] Azar FM , Brandt JC , Miller RH , Phillips BB (2011) Ultra-low-velocity knee dislocations. Am J Sports Med 39, 2170–2174. 21757779 10.1177/0363546511414855

[4] Samuel LT , Rabin J , Jinnah A , et al. (2019) Management of the multi-ligamentous injured knee: An evidence-based review. Ann Jt 4, 4–21.

[5] Holmes CA , Bach BR (1995) Knee dislocations: Immediate and definitive care. Phys Sportsmed 23, 69–82. 10.1080/00913847.1995.11947870 29278152

[6] Shelbourne K , Porter D , Clingman J , McCarroll J , Rettig A (1991) Low-velocity knee dislocation. Orthop Rev 20, 995–1004. 1749665

[7] Reckling FW , Peltier LF (2004) Acute knee dislocations and their complications. Clin Orthop Relat Res 422, 135–141. 10.1097/01.blo.0000131737.72363.7e 15187846

[8] Windsor R , Insall J (1994) Surgery of the knee, 2nd edn. Philadelphia, WB Saunders Company.

[9] Johnson JP , Kleiner J , Klinge SA , McClure PK , Hayda RA , Born CT (2018) Increased incidence of vascular injury in obese patients with knee dislocations. J Orthop Trauma 32, 82–87. 29065033 10.1097/BOT.0000000000001027

[10] Burrus MT , Werner BC , Griffin JW , Gwathmey FW , Miller MD (2016) Diagnostic and management strategies for multiligament knee injuries. JBJS Rev 4, 1. 10.2106/JBJS.RVW.O.00020 27490131

[11] Khakha RS , Day AC , Gibbs J , et al. (2016) Acute surgical management of traumatic knee dislocations – Average follow-up of 10 years. Knee 23, 267–275. 26545616 10.1016/j.knee.2015.09.019

[12] Medina O , Bs GAA , Yeranosian MG , Petrigliano FA , Mcallister DR (2014) Vascular and nerve injury after knee dislocation a systematic review. Clin Orthop Relat Res 472, 2621–2629. 24554457 10.1007/s11999-014-3511-3 PMC4117866

[13] International Monetary Fund (2019) World Economic Outlook – GDP per capita, current prices. Accessed on 15 October 2019.

[14] Dell AJ , Gray S , Fraser R , Held M , Dunn R (2018) Orthopaedic surgeon density in South Africa. World J Surg 42, 3849–3855. 29947987 10.1007/s00268-018-4709-4

[15] LaPrade RF , Chahla J , DePhillipo NN , et al. (2019) Single-stage multiple-ligament knee reconstructions for sports-related injuries: Outcomes in 194 patients. Am J Sports Med 47, 2563–2571. 31381372 10.1177/0363546519864539

[16] Meara JG , Leather AJM , Hagander L , et al. (2015) Global Surgery 2030: Evidence and solutions for achieving health, welfare, and economic development. Lancet 386, 569–624. 25924834 10.1016/S0140-6736(15)60160-X

[17] Dedmond BT , Almekinders LC (2001) Operative versus nonoperative treatment of knee dislocations: A meta-analysis. Am J Knee Surg 14, 33–38. 11216717

[18] Adams R , Jones A , Lefmann S , Sheppard L (2015) Rationing is a reality in rural physiotherapy: A qualitative exploration of service level decision-making. BMC Health Serv Res 15, 121. 25880469 10.1186/s12913-015-0786-3 PMC4383192

[19] Levy BA , Dajani KA , Whelan DB , et al. (2009) Decision making in the multiligament-injured knee: An evidence-based systematic review. Arthroscopy 25, 430–438. 19341932 10.1016/j.arthro.2009.01.008

[20] Mook WR , Miller MD , Diduch DR , Hertel J , Boachie-Adjei Y , Hart JM (2009) Multiple-ligament knee injuries: A systematic review of the timing of operative intervention and postoperative rehabilitation. JBJS 91, 2946–2957. 10.2106/JBJS.H.01328 19952260

[21] Sheth U , Sniderman J , Whelan DB (2019) Early surgery of multiligament knee injuries may yield better results than delayed surgery: A systematic review. J ISAKOS Jt Disord Orthop Sport Med 4, 26–32.

[22] Oro FB , Sikka RS , Wolters B , et al. (2011) Autograft versus allograft: An economic cost comparison of anterior cruciate ligament reconstruction. Arthroscopy 27, 1219–1225. 21820267 10.1016/j.arthro.2011.04.008

[23] Mistry H , Metcalfe A , Colquitt J , et al. (2019) Autograft or allograft for reconstruction of anterior cruciate ligament: A health economics perspective. Knee Surgery Sport Traumatol Arthrosc 27, 1782–1790. 10.1007/s00167-019-05436-z PMC6541574 30874836

[24] Shaerf DA , Pastides P , Sarraf K , Willis-Owen C (2014) Anterior cruciate ligament reconstruction best practice: A review of graft choice. World J Orthop 5, 23. 24649411 10.5312/wjo.v5.i1.23 PMC3952691

[25] Levy BA , Fanelli GC , Whelan DB , et al. (2009) Controversies in the Treatment of Knee Dislocations and Multiligament Reconstruction. J Am Acad Orthop Surg 17, 197–206. 19307669 10.5435/00124635-200904000-00001

[26] Hollis JD , Daley BJ (2005) 10-Year review of knee dislocations: Is arteriography always necessary? J Trauma 59, 672–676. 16361911

[27] Jones R , Smith E , Bone G (1979) Vascular and orthopedic complications of knee dislocation. Surg Gynecol Obstet 149, 554–558. 483133

[28] Kendall RW , Taylor DC , Salvian AJ , O’Brien PJ (1993) The role of qrteriography qn qssessing vascular injuries associated with dislocations of the knee. J Trauma Inj Infect Crit Care 35, 875–878. 10.1097/00005373-199312000-00013 8263986

[29] Stannard JP , Sheils TN , Lopez-Ben RR (2004) Vascular injuries in knee dislocations: The role of physical examination in determining the need for arteriography. J Vasc Surg 40, 1061. 15118031

